# Exogenous surfactant application in a rat lung ischemia reperfusion injury model: effects on edema formation and alveolar type II cells

**DOI:** 10.1186/1465-9921-9-5

**Published:** 2008-01-18

**Authors:** Niels Dreyer, Christian Mühlfeld, Antonia Fehrenbach, Thomas Pech, Sebastian von Berg, Ragi Nagib, Joachim Richter, Thorsten Wittwer, Thorsten Wahlers, Matthias Ochs

**Affiliations:** 1 Department of Anatomy, Division of Electron Microscopy, University of Göttingen, Kreuzbergring 36, D-37075 Göttingen, Germany; 2 Institute of Anatomy, University of Bern, Baltzerstrasse 2, CH-3012 Bern, Switzerland; 3 Clinical Research Group "Chronic Airway Diseases", Department of Internal Medicine (Respiratory Medicine), Philipps-University, Baldingerstrasse, D-35043 Marburg, Germany; 4 Department of Cardiothoracic and Vascular Surgery, Friedrich Schiller University, Erlanger Allee 101, D-07747 Jena, Germany; 5 Department of Cardiothoracic Surgery, University Hospital of Cologne, Kerpener Str. 62, D-50924 Cologne, Germany

## Abstract

**Background:**

Prophylactic exogenous surfactant therapy is a promising way to attenuate the ischemia and reperfusion (I/R) injury associated with lung transplantation and thereby to decrease the clinical occurrence of acute lung injury and acute respiratory distress syndrome. However, there is little information on the mode by which exogenous surfactant attenuates I/R injury of the lung. We hypothesized that exogenous surfactant may act by limiting pulmonary edema formation and by enhancing alveolar type II cell and lamellar body preservation. Therefore, we investigated the effect of exogenous surfactant therapy on the formation of pulmonary edema in different lung compartments and on the ultrastructure of the surfactant producing alveolar epithelial type II cells.

**Methods:**

Rats were randomly assigned to a control, Celsior (CE) or Celsior + surfactant (CE+S) group (n = 5 each). In both Celsior groups, the lungs were flush-perfused with Celsior and subsequently exposed to 4 h of extracorporeal ischemia at 4°C and 50 min of reperfusion at 37°C. The CE+S group received an intratracheal bolus of a modified natural bovine surfactant at a dosage of 50 mg/kg body weight before flush perfusion. After reperfusion (Celsior groups) or immediately after sacrifice (Control), the lungs were fixed by vascular perfusion and processed for light and electron microscopy. Stereology was used to quantify edematous changes as well as alterations of the alveolar epithelial type II cells.

**Results:**

Surfactant treatment decreased the intraalveolar edema formation (mean (coefficient of variation): CE: 160 mm^3 ^(0.61) vs. CE+S: 4 mm^3 ^(0.75); p < 0.05) and the development of atelectases (CE: 342 mm^3 ^(0.90) vs. CE+S: 0 mm^3^; p < 0.05) but led to a higher degree of peribronchovascular edema (CE: 89 mm^3 ^(0.39) vs. CE+S: 268 mm^3 ^(0.43); p < 0.05). Alveolar type II cells were similarly swollen in CE (423 μm^3^(0.10)) and CE+S (481 μm^3^(0.10)) compared with controls (323 μm^3^(0.07); p < 0.05 vs. CE and CE+S). The number of lamellar bodies was increased and the mean lamellar body volume was decreased in both CE groups compared with the control group (p < 0.05).

**Conclusion:**

Intratracheal surfactant application before I/R significantly reduces the intraalveolar edema formation and development of atelectases but leads to an increased development of peribronchovascular edema. Morphological changes of alveolar type II cells due to I/R are not affected by surfactant treatment. The beneficial effects of exogenous surfactant therapy are related to the intraalveolar activity of the exogenous surfactant.

## Background

Despite the beneficial developments in clinical lung transplantation during the last years the postoperative outcome is still impaired by the occurrence (10–25%) of primary graft dysfunction which manifests as acute lung injury (ALI)/acute respiratory distress syndrome (ARDS) [1]. Primary graft dysfunction contributes significantly to early postoperative morbidity and mortality [1-3]. Ischemia-reperfusion (I/R) injury in the course of lung transplantation is the main cause of primary graft dysfunction. I/R injury is associated with severe structural and functional pulmonary alterations, e.g. intraalveolar and interstitial edema or loss of blood-air barrier integrity [4,5]. Among the different pulmonary changes during I/R the inactivation of surfactant or the imbalance of surfactant function are known to be of significant importance in the setting of ALI/ARDS [6-8].

In fact, surfactant is a complex mixture of lipids, mainly saturated phospholipids, and proteins, including four surfactant apoproteins (SP-A, -B, -C, and -D), which is essential for the structural and functional integrity of the lung [9]. In the alveoli, secreted surfactant is present in different morphological forms which are attributed to be of different functional significance [7,10] and, most importantly, it forms a lining layer at the air-liquid interface which prevents the alveolar collapse during expiration [11]. The cells that synthesize, store, secrete and recycle surfactant are the alveolar epithelial type II cells (AE2). Surfactant lipids as well as parts of the surfactant proteins are stored in AE2 in specific organelles, the lamellar bodies [12,13]. Disturbance of the highly regulated homeostasis of pulmonary surfactant may result in severe pulmonary dysfunction leading to ALI/ARDS. Therefore, preservation of AE2 during I/R is of great importance for the outcome of lung transplantation [14].

One of the most promising approaches to improve surfactant function during I/R is the application of exogenous surfactant via the airways [15]. Indeed, the application of exogenous surfactant preparations in the treatment of severe ARDS resulted in an improved pulmonary oxygenation, however, it failed to reduce mortality in large controlled clinical trials of ARDS so far [16,17]. In most clinical situations, it is not possible to predict the development of ALI/ARDS, thus limiting the use of prophylactic exogenous surfactant therapy. However, lung transplantation is a potential clinical situation where this is actually feasible [16]. Indeed, experimental [18-20] as well as initial clinical data [21] suggest that exogenous surfactant therapy of the donor lung mitigates I/R injury.

However, little is known as to whether the beneficial effect of exogenous surfactant is due to an intraalveolar edema reduction and/or due to an enhanced preservation of AE2 and, therefore, the homeostasis of endogenous surfactant. Assessment of pulmonary edema formation in different lung compartments and the ultrastructural integrity of AE2 are important tools with high predictive value in studies investigating lung preservation quality [4,5]. Therefore, we aimed at identifying the effects of exogenous surfactant therapy on the structural alterations induced by the whole sequence of transplantation-related events including lung preservation, ischemic storage and reperfusion. Using a reliable extracorporeal rat lung I/R injury model [22], we established the pre-ischemic intratracheal administration of exogenous surfactant and studied perfusate oxygenation, peak inspiratory pressure and pulmonary vascular resistance during reperfusion. At the end of the protocol, the lungs were prepared for quantitative light and electron microscopical analysis. Stereological estimations included the edema formation in different pulmonary compartments as well as a detailed analysis of the AE2 and their lamellar bodies.

## Methods

### Animals

Fifteen male Sprague-Dawley rats (Crl:CD; Charles River, Sulzfeld, Germany) with a body weight of 430 ± 19 g (mean ± SD) were anaesthesized intraperitoneally with pentobarbital (Nembutal 1 mg/kg body weight) intubated by tracheostomy, and heparinized via the vena cava inferior (100 IU). Parts of the data reported for the control group were previously published in Fehrenbach et al. [23] and are indicated as being so in the tables reporting the results. The animals investigated in the presented study, including the controls, were part of the same randomization process, i.e. they were from the same batch, were housed at the same time under the same conditions, received the same food, etc. All animals received humane care in compliance with the "Guide for the Care and Use of Laboratory Animals" published by the National Institute of Health (NIH publication 85–23, revised 1996). The experiments have been approved by the regional government.

### Study design and tissue preparation

The animals were randomly assigned to three groups (n = 5 per group). The reason for choosing 5 animals per group in a stereological study is that if a parameter is found to change in one direction in all 5 cases, then the probability that this is due to chance is p = (1/2)^5 ^< 0.05, thus making the experiment conclusive [24]. Operation and excision of the heart-lung block were performed as described recently [25]. Lungs immediately fixed in situ before excision served as a control group. In the surfactant treated group, 50 mg/kg body weight of Alveofact (Boehringer, Ingelheim, Germany), a modified natural bovine surfactant, was instilled intratracheally immediately before flush perfusion with the preservation solution. The dose of the surfactant bolus was chosen according to the manufacturer's recommendation for neonatal respiratory distress syndrome.

Lungs from both experimental groups were flushed via the pulmonary artery with 20 ml of cold (4°C) Celsior (IMTIX, Pasteur Mérieux, France) solution and stored for 4 hours at 4°C. Reperfusion for 50 minutes was performed with Krebs-Henseleit-buffer (8.0 ml/min at 37°C) containing bovine red blood cells (hematocrit of 38 to 40%) using a quattro head roller pump (Mod-Reglo-Digital; Ismatec, Zürich, Switzerland). During reperfusion, ventilation with room air at a tidal volume of 5 ml and a rate of 40 breaths per minute was continuously performed. A positive end-expiratory pressure (PEEP) of 3 cm H_2_O was maintained. Fixation and tissue sampling were conducted as described recently [4,26].

Briefly, after flush-perfusion with Krebs-Henseleit buffer via the pulmonary artery, the left lungs were fixed by vascular perfusion ex situ (I/R groups) or in situ (control group) with HEPES-buffered glutardialdehyde/paraformaldeyhde (hydrostatic pressure = 15 cm H_2_O; airway pressure = 12 cm H_2_O). At the end of perfusion, the left main bronchus and pulmonary artery were tightly clamped and the organ was stored in cold fixative until further processing. Lung volume was determined by fluid displacement [27] and systematic uniform random samples of lung tissue were taken and processed according to standard methods [26]. By means of a tissue slicer, each organ was cut into 10 to 12 horizontal slices of 3 mm thickness. Starting with a random number, every other slice was chosen for light or electron microscopy, respectively. For light microscopy, the entire slices were subsequently osmicated, immersed in half-saturated watery uranyl acetate, dehydrated in acetone and embedded in glycol methacrylate. For electron microscopy, a transparent point grid was projected onto the sampled slices. Whenever a grid point hit the cut surface of a lung slice, tissue blocks were excised. By this method 8–10 blocks were obtained from each single lung. The tissue blocks were postfixed in osmium tetroxide, stained en bloc in half-saturated watery uranyl acetate, dehydrated in an ascending acetone series and embedded in araldite. Four of the araldite blocks were randomly sampled for ultrastructural analysis.

### Functional parameters

Hemodynamic and respiratory data were recorded during reperfusion as described previously [28,29]. Perfusate oxygenation (ΔPO_2_), defined as the difference between oxygen tension of the perfusate collected from the left atrium after oxygenation (PO_2ox_) and of the deoxygenated perfusate of the pre-load pool (PO_2deox_), was used to assess the capability of gas exchange. Pulmonary vasculary resistance (PVR) was determined by the standard formula given by Fukuse et al. [22].

### Stereological analysis

The sections were analyzed by established stereological methods [30-32] using an Axioskope light microscope (Zeiss, Oberkochen, Germany) equipped with a computer-based stereology system (Cast-Grid 2.00, Olympus, Denmark) or an EM 900 electron microscope (Zeiss, Oberkochen, Germany). All test fields for stereological analysis were obtained by systematic uniform random sampling.

Volume densities were estimated by point counting and used to calculate the absolute volumes by multiplication of the densities with the corresponding reference volume [33]. Thus, the volumes of lung parenchyma and non-parenchyma as well as their compartments (alveolar air space, alveolar edema, alveolar septa, atelectatic regions and peribronchovascular space) were estimated.

The number-weighted mean volume of AE2 was estimated by using light microscopy and the isotropic uniform random rotator [34] based on single-section disector sampling, i.e. only those AE2 that showed a nucleolus were sampled [30]. At the electron microscopic level, the volumes of nucleus, mitochondria and lamellar bodies were estimated by point counting and multiplication of the densities with the number-weighted mean volume of AE2. The number of lamellar bodies was estimated by the disector method [35] based on sets of two parallel ultrathin sections with a thickness of approximately 100 nm (estimated by the Small fold method according to [33]), the reference and the look-up section. In the reference section, AE2 with a corresponding transect in the look-up section were sampled in a systematic uniform random manner. In this way, electron micrographs representing 35–60 pairs of AE2 were collected per lung. The numerical density of lamellar bodies was estimated by counting the tops of lamellar bodies based on the disector method [35] and afterwards multiplied with the number-weighted mean volume of AE2 to obtain the mean number of lamellar bodies within an AE2.

### Statistics

Differences between the experimental groups were tested for significance with the nonparametric Wilcoxon-Mann-Whitney test. The non parametric repeated measures analysis was used to test if the time course of lung functional parameters differed between the two groups. If so, lung functional parameters at 50 minutes vs. 10 minutes were tested individually for significant differences for each group, if not data were pooled. Stereological data are given as mean (CV), with CV = mean/SD. All statistical analysis and graphic presentations were performed using the software program Statistica 6.1 (Statsoft, Tulsa, USA). p values < 0.05 were considered to be significant.

## Results

### Lung function

**Figure 1:**
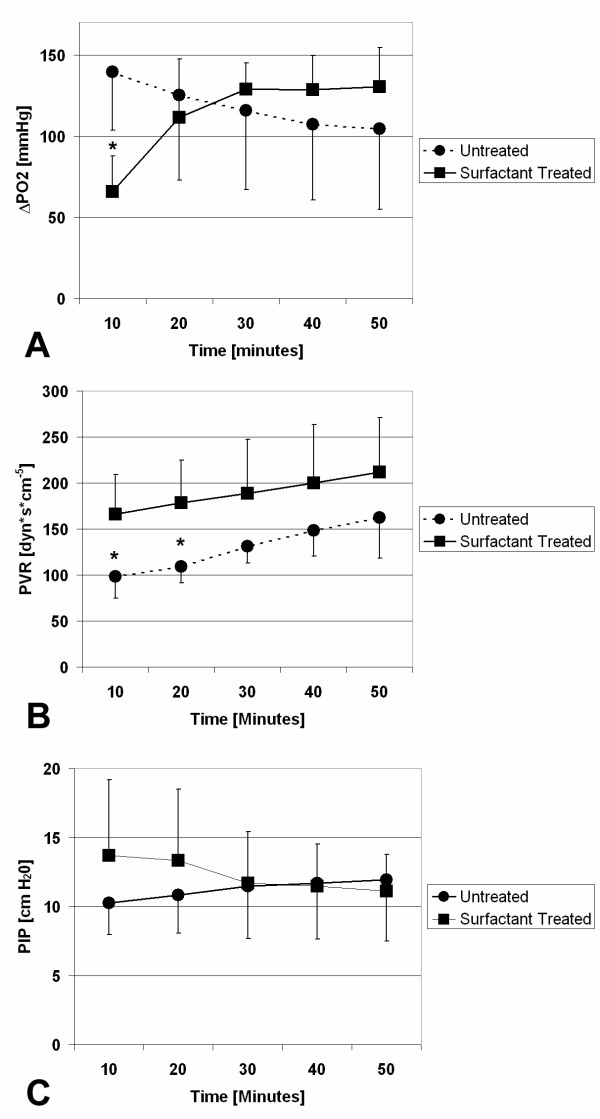
A) Perfusate oxygenation (ΔPO_2_) during reperfusion. After 10 minutes, ΔPO_2 _was decreased in surfactant treated lungs compared to untreated lungs. After 50 minutes, ΔPO_2 _decreased in untreated lungs compared to baseline. B) Pulmonary vascular resistance (PVR) during reperfusion. Repeated measures analysis revealed no significance in the difference of the time course between the groups. The increase in PVR is significant at 50 minutes vs. 10 minutes in both groups. C) Peak inspiratory pressure (PIP) during reperfusion. Whereas alterations in PIP in the surfactant treated group were not significant, the increase in PIP in untreated animals was significant at 50 min vs. 10 minutes. Points and squares represent mean values ± standard deviation. Asterisk indicates statistically significant difference between untreated and surfactant treated group.

After 10 minutes of reperfusion, perfusate oxygenation (ΔPO_2_) was significantly (p < 0.02) decreased in lungs treated with surfactant compared to untreated lungs (Fig. 1A). However, after 30 minutes of reperfusion, ΔPO_2 _in surfactant treated lungs attained similar levels as in untreated lungs. These levels remained unchanged until the end of reperfusion, whereas untreated lungs showed a significant decrease in ΔPO_2 _after 50 minutes of reperfusion compared to baseline. The pulmonary vascular resistance (PVR) was higher in the surfactant group at any time and steadily increased throughout the entire experiment in both experimental groups (Fig. 1B). Initially, the peak inspiratory pressure (PIP) was higher in surfactant treated lungs but continuously decreased with the onset of reperfusion (Fig. 1C), whereas the pressure needed to ventilate untreated lungs increased throughout the entire experiment.

### Qualitative morphological analysis

**Figure 2:**
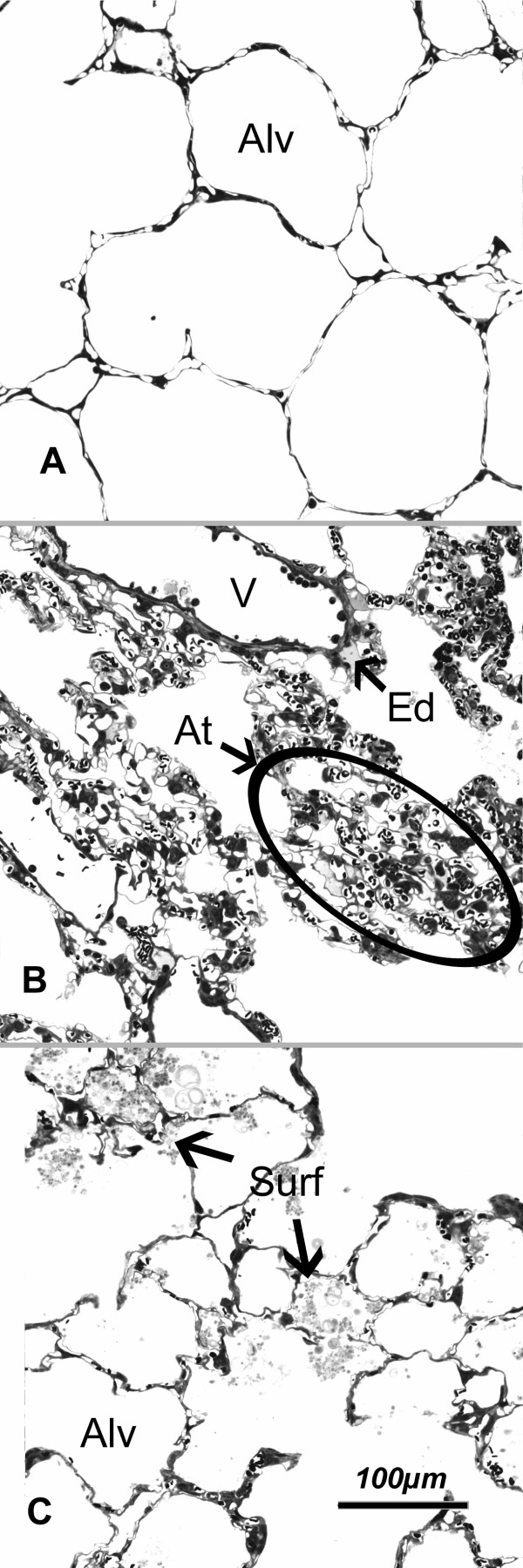
Parenchymal architecture was mostly intact in A) control lung and C) surfactant treated lung subjected to I/R; B) untreated lung subjected to I/R showed regions of alveolar collapse and atelectasis formation. Note the formation of small areas with intraalveolar edema (Ed) in B. After surfactant treatment, numerous small intraalveolar surfactant aggregates were observed. Some larger aggregates almost entirely filled several neighbouring alveoli (Surf) in C. Alv = alveolar lumen; V = vessel; Ed = edema; At = atelectasis; Surf = surfactant.

Control lungs showed the normal parenchymal architecture after vascular perfusion fixation. Alveoli were open and almost free of edema, capillaries were widely open except for some erythrocytes left in the septal capillaries. The parenchyma of the untreated I/R lungs showed numerous atelectatic and dysteletatic regions and small areas of intraalveolar edema, mainly within the atelectases. In the surfactant treated groups, there were no signs of intraalveolar edema or atelectases but airspaces contained large amounts of exogenous surfactant already visible by light microscopy (Fig. 2). In surfactant treated lungs, a prominent swelling of the peribronchovascular space was visible, localized mainly in the proximity of larger vessels. This was observable only to a much lesser extent in untreated I/R lungs and almost absent in control lungs.

**Figure 3:**
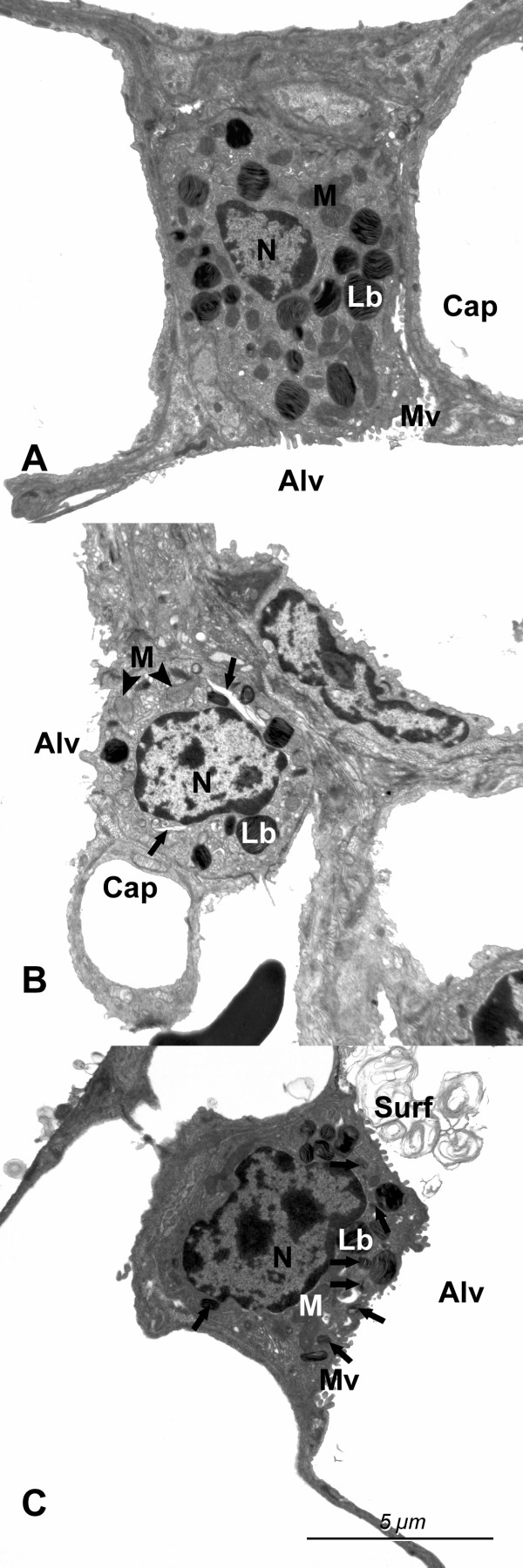
Alveolar type II epithelial cells in A) the control lungs, B) untreated lungs subjected to I/R and C) surfactant treated lungs subjected to I/R. Note the dilations of the endoplasmic reticulum (arrows) in the untreated I/R group and the large amount of small lamellar bodies (arrows) in the surfactant treated group. The mitochondria (M) had a more electron lucent matrix in the untreated I/R group than in the other two groups. Lb = lamellar bodies; Cap = capillary lumen; Alv = alveolar lumen; Surf = surfactant; N = Nucleus.

The ultrastructure of AE2 was normal in control and surfactant treated animals. Only in the untreated I/R group was there occasional dilations of the endoplasmic reticulum and the appearance of mitochondria with electron lucent matrix. The size of the lamellar bodies and their frequency within AE2 varied considerably between the AE2. However, in both experimental groups there were numerous small lamellar bodies in comparison to the control lungs (Fig. 3).

### Stereological analysis

The total lung volume was comparable in all three groups although it tended to be lower in the untreated I/R group (p = 0.12). Open, i.e. non-atelectatic, parenchymal volume was significantly reduced in untreated I/R lungs compared to surfactant treated and control lungs. Only in the untreated I/R group was there a significant volume of atelectatic lung tissue. Intraalveolar edema was present in control and untreated I/R lungs whereas there was only a very small amount in the surfactant treated lungs. The volume of intraalveolar septa was increased in the surfactant treated lungs compared to the controls but not in the untreated I/R lungs. However, adding septal tissue volume of both open and ateletatic regions in the untreated I/R lungs results in similar values of septal tissue volume as in the surfactant treated lungs. The significantly largest volume of the peribronchovascular space was present in the surfactant treated lungs with no differences between control and untreated I/R lungs (Table 1).

**Table 1:** Stereological data on edema formation in parenchymal and non-parenchymal compartments.

	**Control (n = 5)**	**Celsior (n = 5)**	**Celsior/Alveofact (n = 5)**
V(Lung), [mm^3^]	6868 (0.12)	5516 (0.25)	6856 (0.13)
V(Open par, lung), [mm^3^]	6086 (0.12)	4514 (0.25)*	5842 (0.14)†
- V(Air, lung), [mm^3^]	5186 (0.14)	3560 (0.26)*	4679 (0.19)
- V(Ed, lung), [mm^3^]	180 (0.63)	160 (0.61)	4 (0.75)*†
- V(Sept, lung), [mm^3^]	395 (0.23)	445 (0.30)	622 (0.25)*
V(At, lung), [mm^3^]	0	342 (0.90)*	0†
V(Non par, lung), [mm^3^]	782 (0.26)	660 (0.35)	1014 (0.24)
V(Pbv, lung), [mm^3^]	44 (1.25)	89 (0.39)	268 (0.43)*†

Note. All data on edema formation are given as mean (CV). Definition of abbreviations and symbols: V = volume; Open par = Non-atelectatic parenchyma; Air = parenchymal airspace; Ed = alveolar edema; Sept = alveolar septum; At = atelectasis; Pbv = peribronchovascular space; * = p < 0.05 vs. control; † = p < 0.05 vs. Celsior.

AE2 were significantly swollen in both experimental groups as indicated by an increase in the number-weighted mean volume. This was partly due to a significant increase in the volumes of the nucleus and the mitochondria both in surfactant treated and untreated I/R lungs.

The volume of lamellar bodies was slightly but significantly decreased in the untreated I/R lungs compared to controls. Although lamellar body volume was similar in the untreated I/R as in the surfactant treated group, the difference between surfactant treated and control lungs failed to reach significance due to the large variation of data in the surfactant treated group. Despite the lower total volume of lamellar bodies the mean number of lamellar bodies per AE2 was significantly increased in both experimental groups. The number-weighted volume of lamellar bodies revealed that the intracellular surfactant material of the experimental lungs was distributed over a larger number of smaller lamellar bodies than in the control group (Table 2).

**Table 2:** Stereological data on alveolar epithelial type II cells and lamellar bodies.

	**Control (n = 5)**	**Celsior (n = 5)**	**Celsior/Alveofact (n = 5)**
${\bar{\nu }}_{N}$ (AE2), [μm^3^]	323 (0.07) §	423 (0.10)*	481 (0.10)*
V(N, AE2), [μm^3^]	65.0 (0.14) §	116.7 (0.12)*	126.6 (0.13)*
V(M, AE2), [μm^3^]	21.4 (0.15) §	42.4 (0.10)*	48.6 (0.16)*
V (Lb, AE2), [μm^3^]	58.2 (0.09) §	47.3 (0.05)*	47.0 (0.29)
N(Lb, AE2)	93 (0.13) §	189 (0.17)*	242 (0.26)*
${\bar{\nu }}_{N}$ (Lb), [μm^3^]	0.630 (0.09) §	0.255 (0.16)*	0.207 (0.38)*

Note. All data on AE2 and their lamellar bodies are given as mean (CV). Definition of abbreviations and symbols: ${\bar{\nu }}_{N}$ = number-weighted mean volume; V = volume; AE2 = alveolar epithelial type II cells; N = nucleus; M = mitochondria; Lb = lamellar body; § = data from Fehrenbach et al. [23]; * = p < 0.05 vs. control; † = p < 0.05 vs. Celsior.

## Discussion

Although there have been great advances in the clinical outcome of lung transplantation during the past years, primary graft dysfunction showing the clinical aspect of ALI/ARDS is still a significant problem in the early post-transplantation period limiting the success of the whole procedure [3]. Therefore, synergistic approaches for optimization of graft preservation are needed. While great efforts have been made to protect the lung from I/R coming from the vascular side [8,36], preservation from the airway side has come into focus quite recently: Prophylactic exogenous surfactant therapy seems to be a promising tool to enhance the structural and functional preservation of donor lungs during I/R [15]. Experimental lung transplantation has been performed in a variety of animal models using different durations of ischemia and reperfusion and different time points and methods of surfactant application [14,18,19,37-40]. However, it is currently unclear how exogenous surfactant exerts its positive effects in I/R injury. We hypothesized that exogenous surfactant therapy in I/R injury results in reduction of pulmonary edema formation and enhanced ultrastructural integrity of surfactant-producing AE2 and their surfactant-storing lamellar bodies. Stereological and light microscopical investigation of the lung in I/R injury allows to assess the grade of edema in terms of total volumes and to distinguish between different localizations of edema formation (intraalveolar, septal or peribronchovascular), thus enabling a more detailed pathophysiological interpretation than, for example, the determination of wet/dry ratios [5,29,41,42]. Furthermore, combining the high resolution of transmission electron microscopy with stereology gives insight into the quantitative ultrastructure of AE2 and their lamellar bodies and therefore allows to study the effect of I/R and of exogenous surfactant treatment on the endogenous surfactant system [43-45]. The present study is the first evaluation of exogenous surfactant treatment effects in I/R injury on lung structure using a combined light and electron microscopic and design-based stereological approach.

The present study was performed on a reliable isolated lung model that allows to study the consequences of the sequence of transplantation related events including lung preservation, ischemic storage and subsequent reperfusion. However, it needs to be mentioned that our model does not represent a real transplantation model. Therefore, it cannot be evaluated whether the lung injury observed in our study is representative of the clinical situation of ALI/ARDS after transplantation. In particular, our model is not suited for longer observation times and for the investigation of immunomodulatory effects resulting from the interaction between donor and host. In order to evaluate the functional significance of the experimental procedure, we estimated perfusate oygenation, pulmonary vascular resistance and peak inspiratory pressure. Although these measurements provide a comparison between the I/R groups, the interpretation with respect to unchallenged lungs is limited due to the lack of control data as the control lungs were not perfused. Despite these limitations, the animal model is valuable to provide a mechanistic picture of the beneficial effects of exogenous surfactant in I/R injury.

Pulmonary edema is a hallmark of ALI/ARDS and shows a sequential development within the different compartments of the lung [46,47]. The first compartment that shows fluid accumulation is the connective tissue that surrounds the bronchi and larger blood vessels, the peribronchovascular space. In the following, the interstitium of alveolar septa shows an increase in volume which is followed by the flooding of the alveolar lumen by fluid. Since the occurrence of fluid in the alveoli leads to an increase in the oxygen diffusion distance and a decrease in the oxygen diffusion area this type of edema is the functionally most significant one [29]. In the present study, it could be shown that exogenous surfactant therapy effectively decreases the development of intraalveolar edema while peribronchovascular edema is more pronounced than in control and untreated I/R animals. Furthermore, surfactant treatment abolished the development of I/R associated atelectases. These results are in good accordance with the perfusate oxygenation which was initially decreased due to surfactant treatment but gradually increased during reperfusion until a plateau was reached. The initial decrease can be explained by the surfactant bolus which resolves after a period of time. In the untreated rats, perfusate oxygenation decreased during reperfusion, possibly due to the development of intraalveolar edema and atelectases [29].

Although AE2 occupy only a small percentage of alveolar surface area [48] they are essential for pulmonary function. First, they contribute to regeneration of the alveolar epithelium under physiological and pathological conditions. Second, they are the main producers of surfactant and their metabolism also includes surfactant storage, secretion, reuptake and recycling of surfactant components [13]. Damage or dysfunction of this delicate cell type therefore affects the function of the whole lung. Preservation of AE2 ultrastructure, in particular their lamellar bodies, correlated with postoperative outcome in clinical lung transplantation [49]. The present study showed an increase in AE2 volume during I/R which was not different between untreated and surfactant treated lungs and mainly occurred due to a swelling of nuclei and mitochondria. I/R injury led to a small decrease in the volume of lamellar bodies per AE2 which only reached statistical significance in the untreated lungs. Interestingly, the number of lamellar bodies per AE2 was increased in both I/R groups and was not altered by surfactant application. Taken together, I/R led to a similar or slightly decreased volume of lamellar bodies which, however, was distributed over a greater number of smaller lamellar bodies. These changes were observed in response to I/R injury and were not affected by surfactant treatment. To our knowledge, the only morphological investigation on the effects of exogenous surfactant on AE2 and their lamellar bodies in the adult lung was performed by Pinkerton et al. [50]. They observed a decrease in the volume fraction and profile size of lamellar bodies in uninjured rabbit lungs. However, the present study emphasizes the necessity to gain data in absolute terms by design-based stereology [32] as the swelling of AE2 led to a strong decrease in lamellar body volume fraction despite only slight changes in total volume. The occurrence of numerous small lamellar bodies after I/R may hint to an enhanced secretion and beginning synthesis of lamellar bodies during I/R [50].

## Conclusion

In summary, the present study showed that exogenous surfactant treatment decreases the development of intraalveolar edema and atelectases in I/R injury but is associated with a pronounced swelling of the peribronchovascular space. Exogenous surfactant does not influence the I/R induced changes in AE2 ultrastructure and lamellar body content. It is therefore concluded that the beneficial effects of exogenous surfactant application observed in this study are related to the intraalveolar activity of the administered material while the morphological appearance of the intracellular surfactant pool remains unaffected. Future studies will need to systematically address the optimal timing, the best surfactant preparations and possible adjuvants that might further increase the beneficial effects of exogenous surfactant therapy in I/R injury.

## Abbreviations

I/R = ischemia and reperfusion

CE = Celsior group

CE + S = Celsior + Surfactant group

ALI = acute lung injury

ARDS = acute respiratory distress syndrome

AE2 = alveolar epithelial type II cell

PEEP = positive end expiratory pressure

ΔPO_2 _= perfusate oxygenation

PO_2ox _= oxygenated perfusate

PO_2deox _= deoxygenated perfusate

PVR = pulmonary vascular resistance

PIP = peak inspiratory pressure

CV = coefficient of variation

## Competing interests

The author(s) declare that they have no competing interests.

## Authors' contributions

**ND: **performed the stereological analysis, calculated the data and made substantial contribution to the analysis and interpretation of the data and wrote the first draft of the manuscript.

**CM: **made substantial contribution to the analysis and interpretation of the data and wrote major parts of the final manuscript.

**AF: **made substantial contribution to the conception and design of the study and supervised the stereological analysis.

**TP: **performed the animal experiments and made substantial contribution to the analysis and interpretation of the functional data.

**SvB: **performed part of the stereological analysis, calculated the data and contributed to the analysis and interpretation of the stereological data

**RN: **performed the animal experiments and made substantial contribution to the analysis and interpretation of the functional data.

**JR: **contributed to the conception and design of the study, supervised the microscopical analysis and revised the manuscript critically.

**TWi: **contributed to the conception and design of the study, supervised the animal experiments and revised the manuscript carefully.

**TWa: **contributed to the conception and design of the study, supervised the animal experiments and revised the manuscript carefully.

**MO: **made substantial contribution to the conception and design of the study; organized, performed and supervised the microscopical analysis, made substantial contribution to data analysis and interpretation and to the final manuscript.

All authors read and approved the final manuscript.
